# Supplementary material for: HIVprotI: an integrated web based platform for prediction and design of HIV proteins inhibitors
Source: J Cheminform. 2018 Mar 9;10:12. doi: 10.1186/s13321-018-0266-y (PMC5845081; doi:10.1186/s13321-018-0266-y)
Supplement: Supplementary file 2 — Additional file 2. Source code of HIVProtI web server. [file 13321_2018_266_MOESM2_ESM.zip › HIVprotI_Source-code/hivprotI/weka-3-6-6/doc/weka/gui/treevisualizer/Edge.html]

Edge


---


|  |  |  |
| --- | --- | --- |
| **Overview** \| **Package** \| **Class** \| **Tree** \| **Deprecated** \| **Index** \| **Help** \| **Weka's home** | |  |
| **PREV CLASS**   **NEXT CLASS** | **FRAMES**    **NO FRAMES**     **All Classes** |  |
| SUMMARY: NESTED \| FIELD \| CONSTR \| METHOD | DETAIL: FIELD \| CONSTR \| METHOD |  |


---


## weka.gui.treevisualizer Class Edge

```
java.lang.Object
  weka.gui.treevisualizer.Edge
```

---

``` public class Edge extends java.lang.Object ```

This class is used in conjunction with the Node class to form a tree
structure.
This in particular contains information about an edges in the tree.

**Version:**
:   $Revision: 7059 $

**Author:**
:   Malcolm Ware (mfw4@cs.waikato.ac.nz)

---

**Constructor Summary**

`Edge(java.lang.String label,
java.lang.String source,
java.lang.String target)`
  
          This constructs an Edge with the specified label
and parent , child serial tags.


| **Method Summary** | |
| --- | --- |
| `java.lang.String` | `getLabel()`             Get the value of label. |
| `java.lang.String` | `getLine(int n)`             Returns line number *n* |
| `java.lang.String` | `getRsource()`             Get the value of rsource. |
| `java.lang.String` | `getRtarget()`             Get the value of rtarget. |
| `Node` | `getSource()`             Get the value of source. |
| `Node` | `getTarget()`             Get the value of target. |
| `void` | `setRsource(java.lang.String v)`             Set the value of rsource. |
| `void` | `setRtarget(java.lang.String v)`             Set the value of rtarget. |
| `void` | `setSource(Node v)`             Set the value of source. |
| `void` | `setTarget(Node v)`             Set the value of target. |
| `java.awt.Dimension` | `stringSize(java.awt.FontMetrics f)`             This will calculate how large a rectangle using the *FontMetrics* passed that the lines of the label will take up |

**Methods inherited from class java.lang.Object**

`equals, getClass, hashCode, notify, notifyAll, toString, wait, wait, wait`

**Constructor Detail**

### Edge

```
public Edge(java.lang.String label,
            java.lang.String source,
            java.lang.String target)
```

:   This constructs an Edge with the specified label
    and parent , child serial tags.

    **Parameters:**: `label` - The text caption for the edge.: `source` - The ID string for this edges parent.: `target` - The ID string for this edges child.


**Method Detail**

### getLabel

```
public java.lang.String getLabel()
```

:   Get the value of label.

    :   **Returns:**: Value of label.

---


### stringSize

```
public java.awt.Dimension stringSize(java.awt.FontMetrics f)
```

:   This will calculate how large a rectangle using the *FontMetrics*
    passed that the lines of the label will take up

    :   **Parameters:**: `f` - The size information for a particular Font **Returns:**: A Dimension containing the size and width of the text

---


### getLine

```
public java.lang.String getLine(int n)
```

:   Returns line number *n*

    :   **Parameters:**: `n` - The number of the line requested **Returns:**: The string for the line number or NULL if it didn't exist

---


### getRsource

```
public java.lang.String getRsource()
```

:   Get the value of rsource.

    :   **Returns:**: Value of rsource.

---


### setRsource

```
public void setRsource(java.lang.String v)
```

:   Set the value of rsource.

    :   **Parameters:**: `v` - Value to assign to rsource.

---


### getRtarget

```
public java.lang.String getRtarget()
```

:   Get the value of rtarget.

    :   **Returns:**: Value of rtarget.

---


### setRtarget

```
public void setRtarget(java.lang.String v)
```

:   Set the value of rtarget.

    :   **Parameters:**: `v` - Value to assign to rtarget.

---


### getSource

```
public Node getSource()
```

:   Get the value of source.

    :   **Returns:**: Value of source.

---


### setSource

```
public void setSource(Node v)
```

:   Set the value of source. And then call v.addChild to add the edge to
    the Node.

    :   **Parameters:**: `v` - Value to assign to source.

---


### getTarget

```
public Node getTarget()
```

:   Get the value of target.

    :   **Returns:**: Value of target.

---


### setTarget

```
public void setTarget(Node v)
```

:   Set the value of target. And then call v.addParent to add the edge to
    the Node.

    :   **Parameters:**: `v` - Value to assign to target.


---


|  |  |  |
| --- | --- | --- |
| **Overview** \| **Package** \| **Class** \| **Tree** \| **Deprecated** \| **Index** \| **Help** \| **Weka's home** | |  |
| **PREV CLASS**   **NEXT CLASS** | **FRAMES**    **NO FRAMES**     **All Classes** |  |
| SUMMARY: NESTED \| FIELD \| CONSTR \| METHOD | DETAIL: FIELD \| CONSTR \| METHOD |  |


---
